# Non-cell autonomous promotion of astrogenesis at late embryonic stages by constitutive YAP activation

**DOI:** 10.1038/s41598-020-63890-z

**Published:** 2020-04-27

**Authors:** Dasol Han, Mookwang Kwon, Sun Min Lee, Samuel J. Pleasure, Keejung Yoon

**Affiliations:** 1 College of Biotechnology and Bioengineering, Sungkyunkwan University, Suwon, Gyeonggi-do 16419 South Korea; 2 Department of Neurology, University of California San Francisco, San Francisco, California USA

**Keywords:** Glial development, Gliogenesis

## Abstract

Although astrocytes have gained increased recognition as an important regulator in normal brain function and pathology, the mechanisms underlying their genesis are not well understood. In this study, we show that constitutive YAP activation by *in utero* introduction of a non-degradable form of the YAP gene (YAP 5SA) causes productive GFAP^+^ cell generation at late embryonic periods, and this activity is nuclear localization- and TEAD transcription factor-dependent. Moreover, we found that the GFAP^+^ cells were not YAP 5SA-expressing cells themselves but cells in the vicinity *in vivo*. Conditioned medium prepared from YAP 5SA-expressing cells induced GFAP^+^ cell production *in vitro*, suggesting that a soluble factor(s) was mediating the astrogenic activity of YAP 5SA. Indeed, YAP 5SA expression greatly increased CNTF and BMP4 transcription in neural progenitor cells, and a neutralizing antibody against CNTF reduced the astrogenic effects of YAP 5SA-conditioned medium. Furthermore, the YAP 5SA-expressing cells were identified as FN1^+^ mesenchymal cells which are responsible for the precocious astrogenesis. These results suggest a novel molecular mechanism by which YAP activation can induce astrogenesis in a non-cell autonomous manner.

## Introduction

Astrocytes are multifunctional players in many aspects of brain physiology. In addition to basic structural and metabolic support for neurons, astrocytes are known to regulate synaptic transmission^1^, vasomodulation^2^, formation and maintenance of the blood–brain barrier^3^, and long-term potentiation^4^. Several studies have reported that, under pathological conditions, astrocytes are required for nervous system repair^5^, and dysfunction is linked with certain psychiatric disorders such as the autism spectrum disorders and schizophrenia^6,7^.

During mammalian cortical development, neural stem cells differentiate into neurons in mid-neurogenic stages, and then into astrocytes around the birth date^8^. Although this temporal window of neurogenic-to-astrogenic transition was well recognized, the cell fate-regulatory mechanisms which trigger the intrinsic changes toward astrogenesis are still not fully understood. Cell-fate determination of various kinds of progenitors is often regulated by ligand and cell surface receptor interactions which transduce extracellular signals to the nucleus to induce the transcription of specific fate-determining genes. Interleukin 6 (IL6)-related ligands such as cardiotrophin 1 (CT1)^9^, leukemia inhibiting factor (LIF)^10^, ciliary neurotrophic factor (CNTF)^11^, neuropoietin (NP)^12^ and cardiotrophin-like cytokine (CLC)^13^ have been identified as astrogenic cytokines which bind to their corresponding receptor complexes to sequentially phosphorylate JAK and STAT3 leading to STAT3 homodimer formation. TGFβ and BMPs bind and activate the type I and type II receptors resulting in phosphorylation of regulatory SMADs and association with the cofactor SMAD4^14^. Dimerized STAT3 and SMADs each directly bind to the glial fibrillary acidic protein (GFAP) promoter, one of the most well-known astrocyte markers, and increase its expression. In addition, astrogliosis, which produces GFAP^+^ cells under pathological conditions such as tumor and various injuries in the adult brain, is also reported to use the same ligands and receptors as those used in normal embryonic astrogenesis^15–17^.

The Hippo pathway is an evolutionarily conserved signaling pathway that governs cell proliferation and organ growth^18^. Yes-associated protein (YAP) is the core transcriptional coactivator of the mammalian Hippo pathway. Upon Hippo pathway activation, YAP is phosphorylated by upstream MST1/2 and LATS1/2 kinases resulting in cytosolic sequestration and proteosomal degradation^19^. When the upstream kinases are inactive, unphosphorylated YAP translocates into the nucleus and acts as a transcriptional coactivator of several partner transcription factors such as p73, RUNX2 and TEAD^20^. Due to its cell-cycle promoting activity, YAP-related brain studies have mainly focused on the regulation of neural stem cell proliferation. For example, YAP was consistently identified as a positive regulator for neural stem cell pool expansion in the developing chick^21^, frog^22^ and mouse models^23^. In this regard, it is of note that YAP was recently shown to be necessary for BMP2-induced astrogenesis in a conditional YAP knockout model^24,25^.

In this study, we show that constitutive YAP activation induces precocious GFAP^+^ cell production at late embryonic stages in a non-cell autonomous fashion using gain-of-function experiments based on retroviral transduction. Furthermore, we provide evidence that soluble factors such as CNTF contribute to the outcomes of YAP activation to induce astrogenesis. Our study provides a potential mechanism by which a high level of YAP activation regulates astrocyte generation from progenitor cells.

## Results

### Constitutive YAP activation produces SOX2^−^ cell clusters in the ventricular zone (VZ) at late embryonic periods

**Figure 1:**
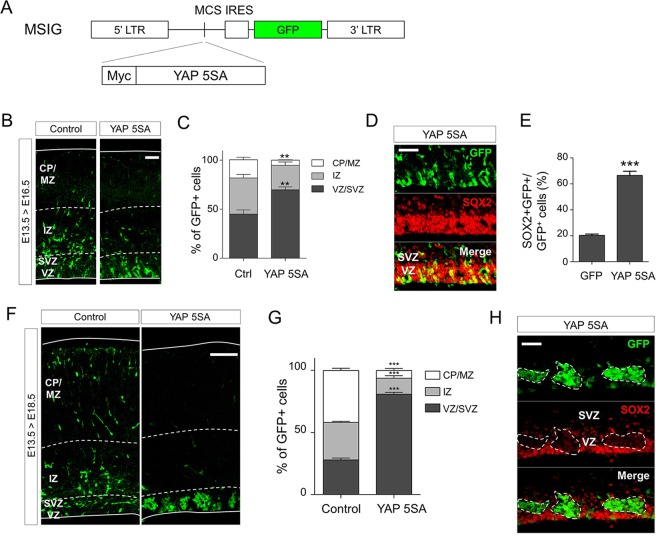
Constitutive YAP activation forms SOX2^−^ cell clusters in the VZ at E18.5. (**A**) Schematic representation of the retroviral vector MSIG used in this study. Internal ribosome entry site (IRES) allows bicistronic expression of YAP 5SA and GFP, and MSIG expressing only GFP without an insert gene was used as a control. LTR, long terminal repeat; MCS, multicloning site. (**B, D**) Fluorescent microscopy of coronal sections of E16.5 embryonic brains that were intraventricularly injected at E13.5 with retroviral vectors expressing YAP 5SA. Gene-transferred cells were labeled with (**B**) anti-GFP antibody alone, or (**D**) a combination of anti-GFP (green) and anti-SOX2 (red) antibodies. (**F, H**) E18.5 brains injected at E13.5 were labeled using (**F**) only anti-GFP or (**H**) anti-GFP (green) and anti-SOX2 (red) primary antibodies. (**C, E, G**) Quantification of (**B, D, F**). Scale bars, 50 μm for (**B, D, H**) and 100 μm for (**F**). LV, lateral ventricle; VZ, ventricular zone; SVZ, subventricular zone; IZ, intermediate zone; CP, cortical plate; MZ, marginal zone. Error bars represent SD. Student’s *t*-test was used to determine statistical significance. **P < 0.01, ***P < 0.001; n ≥ 3.

To address the effects of YAP gain-of-function in the developing brain, we used a YAP mutant, YAP 5SA, which bears serine to alanine substitutions in all five HXRXXS YAP sites and is thus no longer inactivated by the upstream Hippo signaling kinase LATS^26^. The retroviral vector MSIG^27^, bicistronically expressing YAP 5SA and GFP (Fig. 1A), was injected into embryonic day 13.5 (E13.5) mice telencephalic ventricles *in utero* and the cortical distribution of the transduced cells was assessed at E16.5, 3 days posttransduction. This experimental setting resulted in increased YAP 5SA^+^ cell localization in the VZ (Fig. 1B,C). The YAP 5SA-expressing cells in the VZ also stained with an antibody against SOX2, a neural stem cell marker (Fig. 1D). These data collectively imply that YAP activation maintains neural stem cell characteristics at mid-neurogenic periods. We extended our observations to late neurodevelopmental stages by analyzing the effects of YAP activation at E18.5. At E18.5, 5 days posttransduction, YAP 5SA-expressing cells formed clusters, and they were still detected in the VZ (Fig. 1F,G). However, notably, almost complete loss of SOX2 expression was observed in these cell clusters (Fig. 1H). These data suggest that strong YAP activation can lead to dramatically different outcomes in the developing brain, maintenance of the SOX2^+^ neural stem cell pool or formation of SOX2^−^ cell clusters in the VZ, depending on the embryonic stages.

### Constitutive YAP activation induces massive GFAP^+^ cell production at late embryonic periods in a non-cell autonomous manner

**Figure 2:**
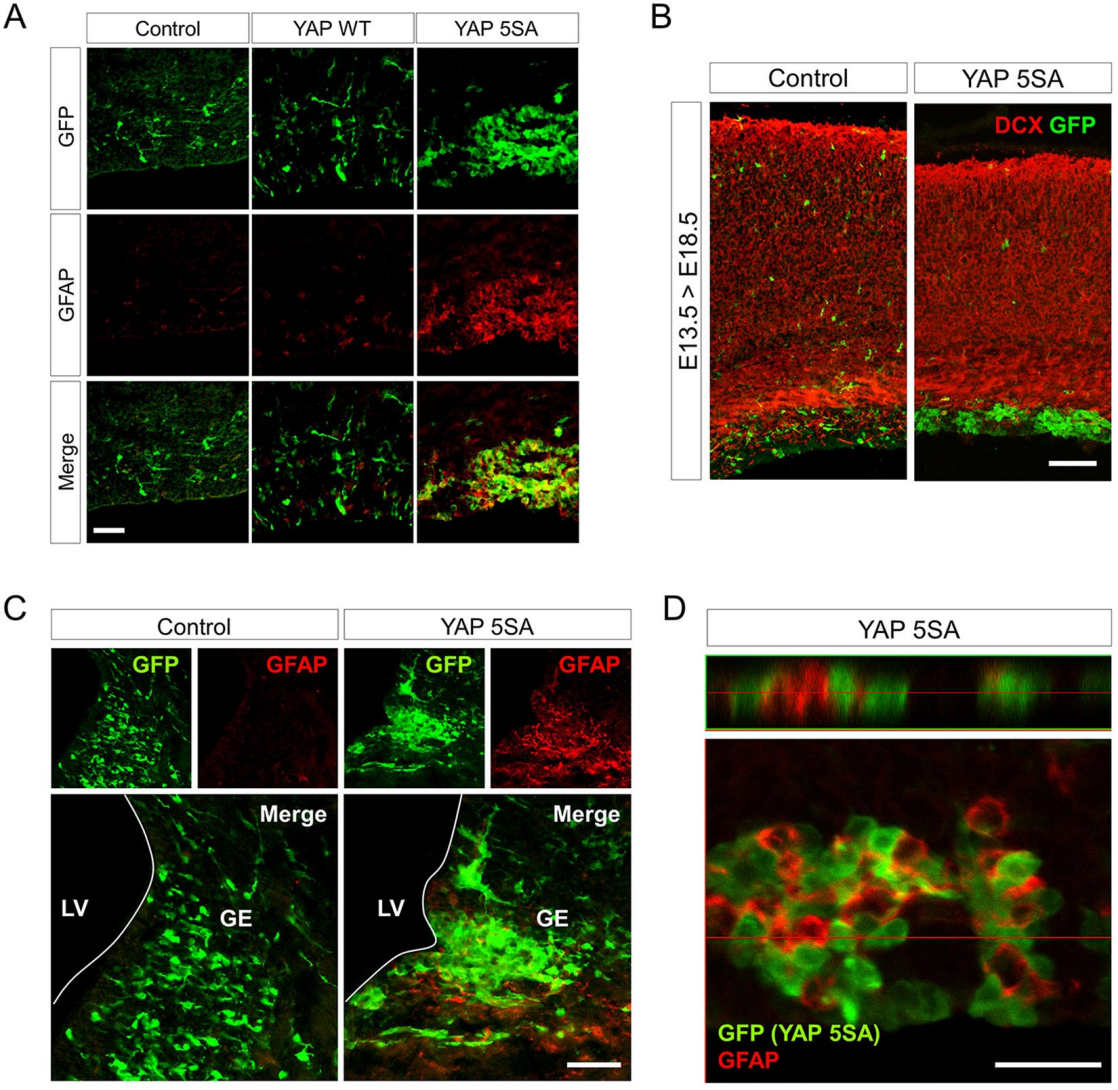
Constitutive YAP activation induces productive GFAP^+^ cell generation at late embryonic periods in a non-cell autonomous fashion. (**A, B, D**) Neocortex or (**C**) ganglionic eminence (**G, E**) regions of E18.5 brains transduced with YAP 5SA retroviruses at E13.5 were double-labeled with the indicated antibodies. (**D**) Single plane confocal image of YAP 5SA-transduced E18.5 neocortex stained with anti-GFP (green) and anti-GFAP (red) antibodies, and the corresponding 14 μm z-stack view (top) at the position of the red line. Scale bars, 25 μm for (**D**), 50 μm for (**A, C**), and 100 μm for (**B**). LV, lateral ventricle.

Next, we performed immunofluorescence assays to determine the characteristics of the SOX2^−^ cell clusters in the VZ at E18.5 induced by YAP 5SA expression. At first glance, a majority of cells in the YAP 5SA-induced cell clusters appeared to be GFAP^+^ astrocytes which are not productively generated at these developmental stages (Fig. 2A), and they did not stain with antibodies against other neural cell type markers such as doublecortin (Dcx, a marker of newly born neurons) (Fig. 2B). Interestingly, unlike YAP 5SA, wild-type YAP overexpression did not induce either cell clusters in the VZ or GFAP^+^ cell production (Fig. 2A). In addition to the cortex, YAP 5SA expression in the ganglionic eminence (GE) region also produced massive GFAP^+^ cells (Fig. 2C). However, GFAP^+^ GFP^−^ cells were readily detected in the GE (Fig. 2C) implying that the GFAP^+^ cells in the GE were not YAP 5SA-expressing cells themselves. These results led us to perform a closer examination of the immunostaining of the cortical region by confocal microscopy, and we observed mutually exclusive expression patterns of YAP 5SA and GFAP in the cortical region (Fig. 2D), again indicating that the GFAP^+^ cells in the YAP 5SA-induced cell clusters were not YAP 5SA^+^ cells, but neighboring cells in close contact. Taken together, these data suggest that strong constitutive activation of the YAP signaling pathway can accelerate astrocyte production in a non-cell autonomous fashion at late developmental stages.

### YAP 5SA induces astrogenesis by secreting heat-labile soluble factor(s)

**Figure 3:**
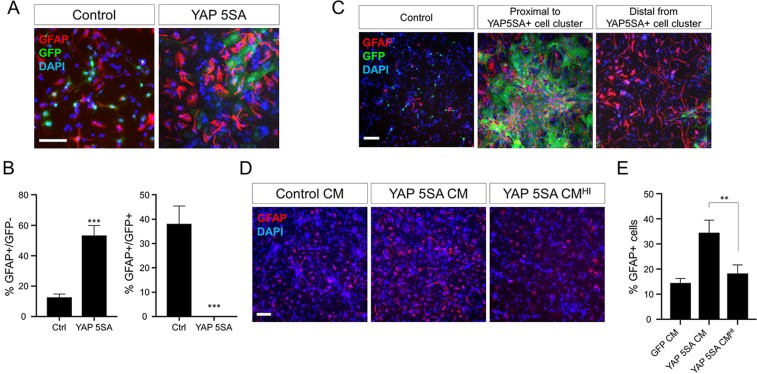
Heat-labile soluble factor(s) mediates YAP 5SA-induced astrogenesis *in vitro*. (**A**) Immunostaining using anti-GFAP after *in vitro* differentiation of co-cultured cells. E13.5 neural progenitor cells transduced with YAP 5SA retroviruses were mixed with untransduced neural progenitor cells at a ratio of 1:5 (transduced:untransduced) and then cultured in differentiation medium for 3 days. Quantification of (**A**) is shown in (**B**). (**C**) GFP (green) and GFAP (red) double immunostaining of cells differentiated under the same experimental conditions as (**A**). (**D**) Untransduced E13.5 neural progenitor cells were cultured in differentiation medium prepared by mixing conditioned medium (CM) of YAP 5SA-transduced neural progenitor culture and fresh differentiation medium in a 1:1 ratio. CM^HI^, heat-inactivated (56 °C for 30 min) CM. (**E**) Quantification of (**D**). The DAPI nuclear counterstain is shown in blue in (**A, D**). Scale bars, 100 μm for (**A, D**), and 200 μm for (**C**). Student’s *t*-test was used to determine statistical significance. **P < 0.01, ***P < 0.001; n ≥ 3.

The ability of YAP 5SA to increase astrogenesis was further examined by an *in vitro* differentiation assay. E13.5 neural progenitors were infected with YAP 5SA retroviral vectors, mixed with untransduced neural progenitor cells at a ratio of 1:5 (transduced:untransduced) and incubated in differentiation medium. As shown in Fig. 3A, YAP 5SA transduction greatly increased GFAP^+^ cell production. In addition, GFAP^+^ cells were found evenly throughout the culture dish, up to the region distal to the GFP^+^ cells (Fig. 3C). These results are reminiscent of *in vivo* effects of YAP 5SA and indicate that soluble factor(s) may mediate the astrogenic effects of YAP 5SA. As expected, conditioned medium from YAP 5SA-transduced neural progenitor cell cultures was sufficient to enhance astrogenesis, and heat-treatment efficiently abrogated the astrogenesis-promoting activity of the conditioned medium (Fig. 3D,E). However, YAP 5SA-expressing cells did not appear to have neural cell morphology (green cells in right panel of Fig. 3C). These data collectively suggest that YAP 5SA expression can induce astrogenesis in a non-cell autonomous fashion *in vitro* as seen under *in vivo* conditions, presumably by inducing heat-labile paracrine factor expression.

### YAP 5SA induces astrogenesis in a nuclear localization-dependent manner

**Figure 4:**
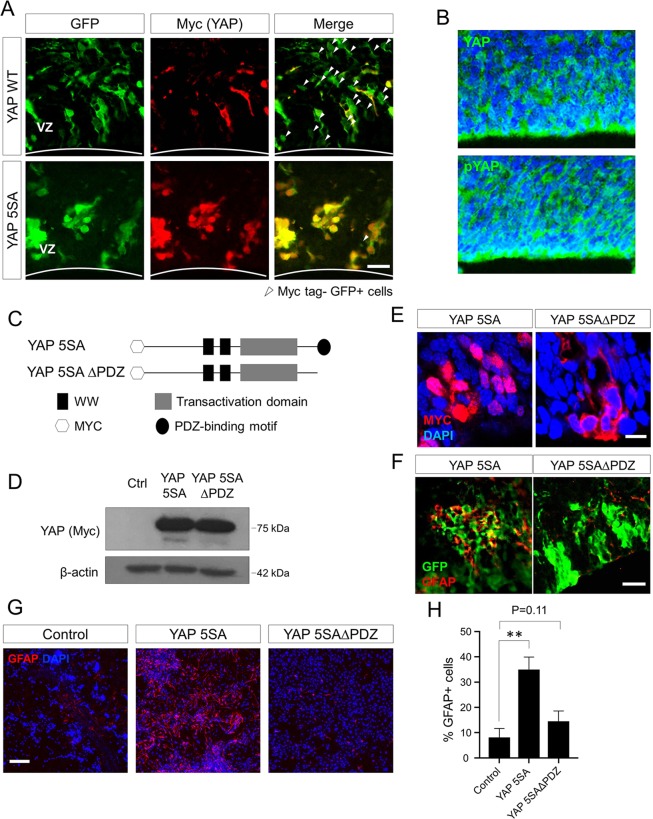
The ability of YAP 5SA to induce astrogenesis is nuclear localization-dependent. (**A**) Neocortical regions of E18.5 brains injected with YAP retroviruses at E13.5 were double-labeled with anti-GFP (green) and anti-Myc tag (red) antibodies. White arrowheads indicate GFP^+^/Myc tag^−^ cells (**B**) Expression pattern of endogenous YAP (top) and phosphorylated form of YAP proteins (bottom) in the VZ at E14.5 were analyzed by immunostaining. (**C**) Schematic diagram showing the domain structures of YAP 5SA. (**D**) Expression of Myc-tagged YAP 5SA and PDZ-binding motif-deleted YAP 5SA (YAP 5SAΔPDZ) genes in transduced HEK 293 T cells was confirmed by Western blotting. (**E, F**) E18.5 brains transduced with YAP 5SA retroviruses at E13.5 were harvested and immunostained using (**E**) anti-Myc tag antibody to test nuclear localization, or (**F**) combination of anti-GFP and anti-GFAP antibodies to test astrogenic ability. (**G**) Immunostaining using anti-GFAP antibody after an *in vitro* differentiation assay. E13.5 neural progenitor cells transduced with YAP retroviruses were mixed with untransduced neural progenitor cells at a ratio of 1:5 (transduced: untransduced) and then cultured in differentiation medium for 3 days. Quantification is shown in (**H**). The DAPI nuclear counterstain is shown in blue in (**B, E, G**). Scale bars, 10 μm for (**E**), 25 μm for (**A, F**), 50 μm for (**B**), and 200 μm for (**G**). Student’s *t*-test was used to determine statistical significance. **P < 0.01; n ≥ 3.

Unlike YAP 5SA, wild-type YAP overexpression did not show strong astrogenic effects at E18.5 *in vivo*. Immunolabeling assays revealed that a large fraction of cells transduced with Myc-tagged wild-type YAP-expressing retroviruses were Myc tag^−^, whereas the most GFP^+^ cells were Myc tag^+^ in the YAP 5SA-Myc transduced samples (Fig. 4A). Considering that the GFP expression assures successful retroviral gene transfer (Fig. 1A), and the Myc signals are proportional to the amount of the exogenously expressed YAP proteins, the levels of overexpressed wild-type YAP proteins were presumed to be reduced after transduction into the brain. The low levels of the exogenously introduced wild-type YAP proteins is likely due to a high level of YAP-phosphorylating activity in the VZ of the developing brain. Indeed, immunolabeling of untransduced E14.5 cortical sections using anti-YAP and anti-phospho-YAP antibodies produced similar staining patterns, with strong signals in the VZ (Fig. 4B). These results suggest that endogenous YAP is expressed in the VZ but mostly exists in a phosphorylated form due to Hippo kinase activity, eventually leading to a low amount of YAP proteins in the nucleus. These data also imply that different levels of proteins in the nucleus may explain the distinct strengths of astrogenic activity between wild-type YAP and YAP 5SA proteins. To test this possibility, we generated a YAP 5SA derivative lacking the PDZ-binding motif, YAP 5SAΔPDZ (Fig. 4C), which is known to be important for nuclear translocation of YAP proteins^28^. Western blot and immunofluorescence microscopy analyses, respectively, showed comparable expression of YAP 5SA and YAP 5SAΔPDZ in HEK 293 T cells *in vitro* (Fig. 4D), but clear nuclear exclusion of YAP 5SAΔPDZ proteins in the VZ *in vivo* (Fig. 4E). As hypothesized, PDZ-binding motif deletion resulted in dramatic loss in astrogenic activity of YAP 5SA in both *in vivo* (Fig. 4F) and *in vitro* (Fig. 4G,H) conditions. These data demonstrate that nuclear localization plays a critical role in YAP 5SA-induced astrogenesis.

### YAP 5SA-mediated transcriptional activation is associated with astrogenic activity

**Figure 5:**
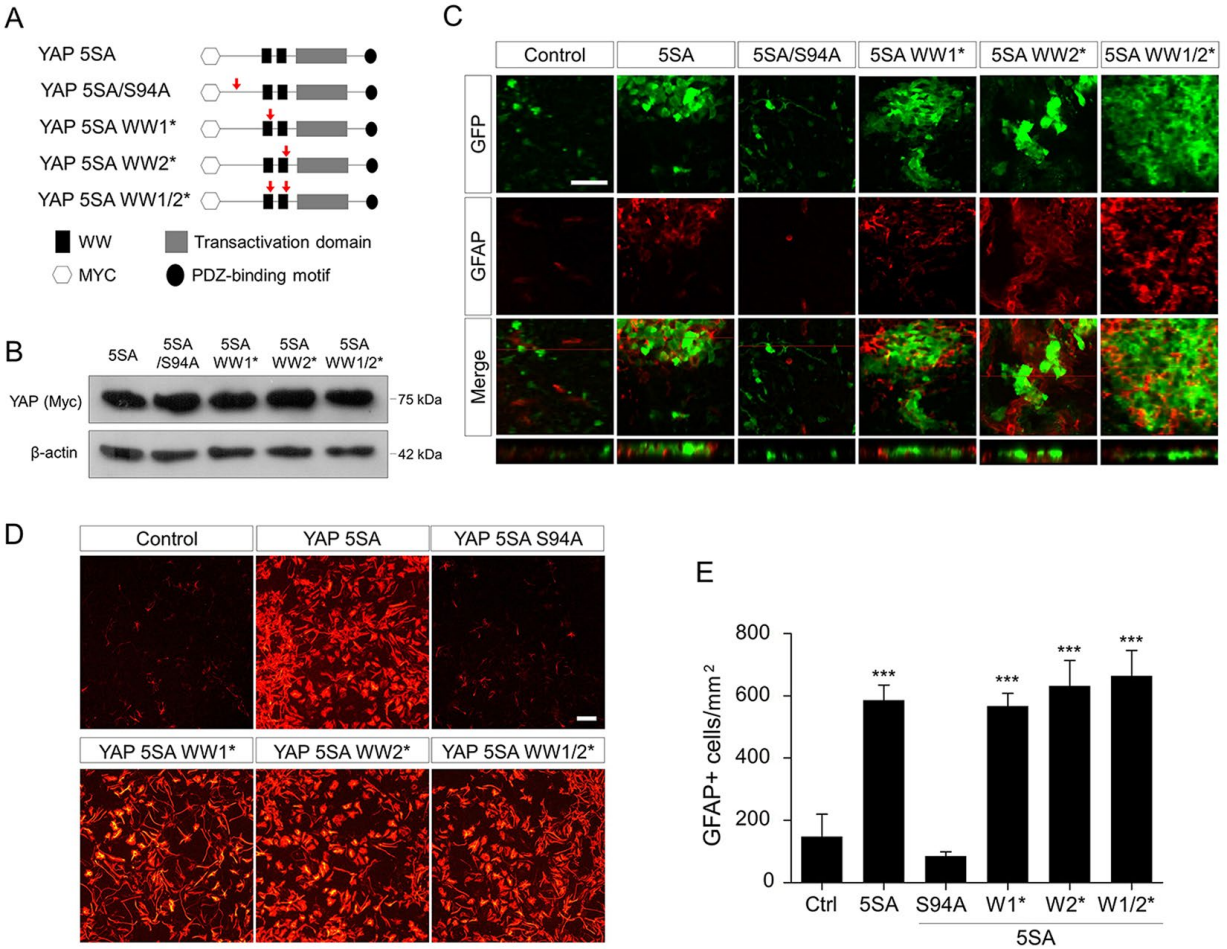
TEAD-dependent transcriptional activation is associated with astrogenic activity of YAP 5SA. (**A**) Schematic diagram showing the location of mutations (red arrows) disrupting interaction with TEAD family transcription factors and WW domain functions in the YAP gene. (**B**) Expression of Myc-tagged YAP 5SA derivative mutants in transduced HEK 293 T cells was confirmed by Western blotting using an anti-Myc tag antibody. (**C**) Confocal microscopy images of wild-type and mutant YAP 5SA-transduced E18.5 neocortices stained with anti-GFP (green) and anti-GFAP (red) antibodies, and the corresponding Z-stack view (bottom) at the position of the red line. (**D**) An *in vitro* differentiation assay was performed after mixing retrovirally transduced E13.5 primary neural progenitor cells with untransduced cells at a ratio of 1:5, and GFAP immunostaining was carried out at 3 days postdifferentiation. (**E**) Quantification of (**D**). Scale bars, 25 μm for (**C**) and 100 μm for (**D**). Student’s *t*-test was used to determine statistical significance. ***P < 0.001; n ≥ 3.

The importance of nuclear localization in YAP 5SA-induced astrogenesis suggests that the astrogenic properity of YAP 5SA is highly likely to be associated with transactivating ability. YAP acts as a transcriptional co-activator of several PPXY motif-bearing transcription factors such as AP2, C/EBPα, c-JUN, OCT-4, p73, and RUNX2 through WW domains^20^. YAP also binds to TEA domain family transcription factors (TEADs) in a WW domain-independent manner^20^. To determine if the transactivational ability of YAP 5SA is required, and if it is, which partner transcription factor is necessary for YAP 5SA-driven astrogenesis, we introduced point mutations into YAP 5SA by site-directed mutagenesis (Fig. 5A). The serine at position 94 was changed to alanine (S94A) to disrupt the interaction of YAP with the TEAD family transcription factors^29^. In addition, the amino acid residues W199QDP202 and W258LDP261 were individually or simultaneously mutated to A199QDA202 and A258LDA261 to impair the WW1 and WW2 domain functions, respectively^30^. Western blot analysis showed that all mutant proteins were produced at comparable levels to the YAP 5SA proteins (Fig. 5B). When these mutants were transduced into the brain, only the S94A mutant showed a great loss of astrogenesis-inducing activity, whereas the WW1, WW2, and WW1/2 mutants behaved like YAP 5SA (Fig. 5C). Consistently, *in vitro* differentiation assay also revealed that only the S94A mutant failed to promote differentiation of neural progenitor cells into GFAP^+^ cells (Fig. 5D,E). These data suggest that YAP 5SA facilitates astrogenesis by TEAD-dependent transcriptional activation.

### Constitutive YAP activation induces astrogenesis by increasing CNTF expression

**Figure 6:**
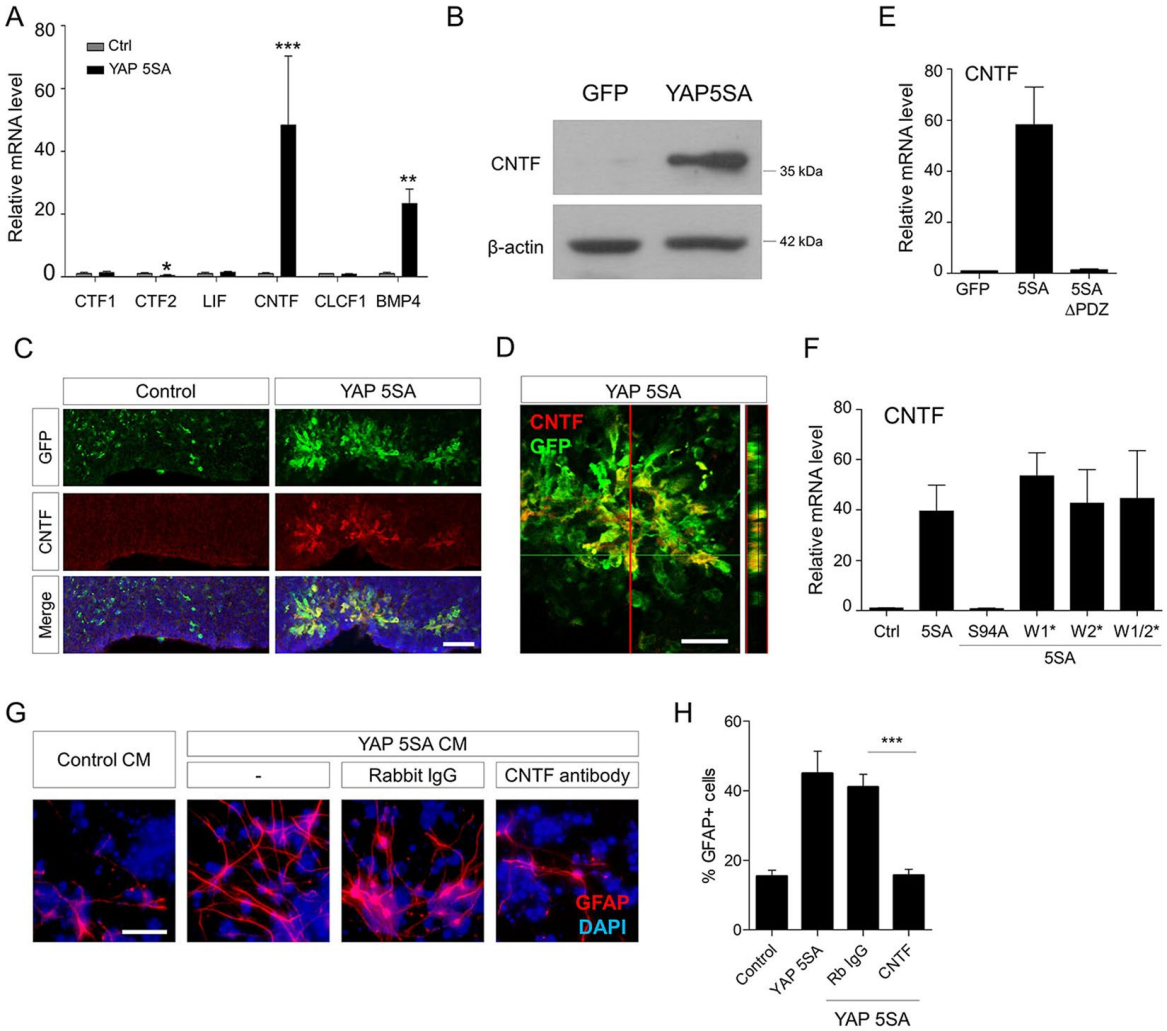
YAP 5SA-expressing cells produce CNTF. (**A**) E13.5 primary neural progenitor cells were transduced with retroviral vectors expressing YAP 5SA and incubated in differentiation medium. After 2 days, mRNA expression levels of indicated astrogenesis-related ligand genes were measured by qPCR. (**B**) Western blot analysis of YAP 5SA-transduced neural progenitor cell lysates using an anti-CNTF antibody. (**C**) E18.5 brains transduced with YAP 5SA retroviruses at E13.5 were harvested and double-immunostained using anti-GFP (green) and anti-CNTF (red) antibodies. (**D**) 14 μm z-stack view (right) at the position of the red line. (**E, F**) The ability of indicated YAP 5SA mutants to induce CNTF expression was tested by qPCR under the same experimental conditions as in (**A**). (**G**) Untransduced E13.5 neural progenitor cells were cultured in the differentiation medium containing conditioned medium of YAP 5SA-transduced neural progenitor culture with or without neutralizing antibodies against CNTF. (**H**) Quantification of (**F**). Scale bars, 25 μm for (**D**), 50 μm for (**C**), and 100 μm for (**G**). Student’s *t*-test was used to determine statistical significance. *P < 0.05, **P < 0.01, ***P < 0.001; n ≥ 3.

To identify the astrogenic determinant(s) associated with YAP 5SA expression, we measured mRNA levels of several ligand genes which are known to be involved in astrogenesis. Quantitative real-time polymerase chain reaction (qPCR) analysis showed that YAP 5SA greatly increased CNTF and BMP4 expression up to 50 and 30 fold, respectively (Fig. 6A). Western blot analysis confirmed increased CNTF protein levels in the YAP 5SA-transduced cell lysates (Fig. 6B). Because BMP4 was reported to be a target gene of transcriptional coactivator with PDZ-binding motif (TAZ) in the breast cancer cells^31^, we focused on the CNTF in this study. Confocal microscopy showed that YAP 5SA-expressing cells were clearly colabeled by anti-CNTF antibody *in vivo* (Fig. 6C,D). Consistent with the *in vivo* GFAP immunolabeling results, YAP 5SAΔPDZ and S94A mutants did not induce CNTF expression, indicating that nuclear localization activity and TEAD-dependent transactivation are associated with increase of CNTF expression, and that CNTF may mediate the astrogenic ability of YAP 5SA (Fig. 6E,F). The importance of CNTF expression in YAP 5SA-induced astrogenesis was further tested by *in vitro* differentiation assays using a CNTF-neutralizing antibody. As shown in Fig. 6G,H, enhanced differentiation of neural progenitor cells into GFAP^+^ cells by YAP 5SA-conditioned media was reduced by CNTF neutralization. These results suggest CNTF is a non-cell autonomous factor mediating YAP 5SA-induced astrogenesis.

### YAP 5SA expression induces generation of fibronectin 1-positive (FN1^+^) mesenchymal cells in a cell autonomous fashion

**Figure 7:**
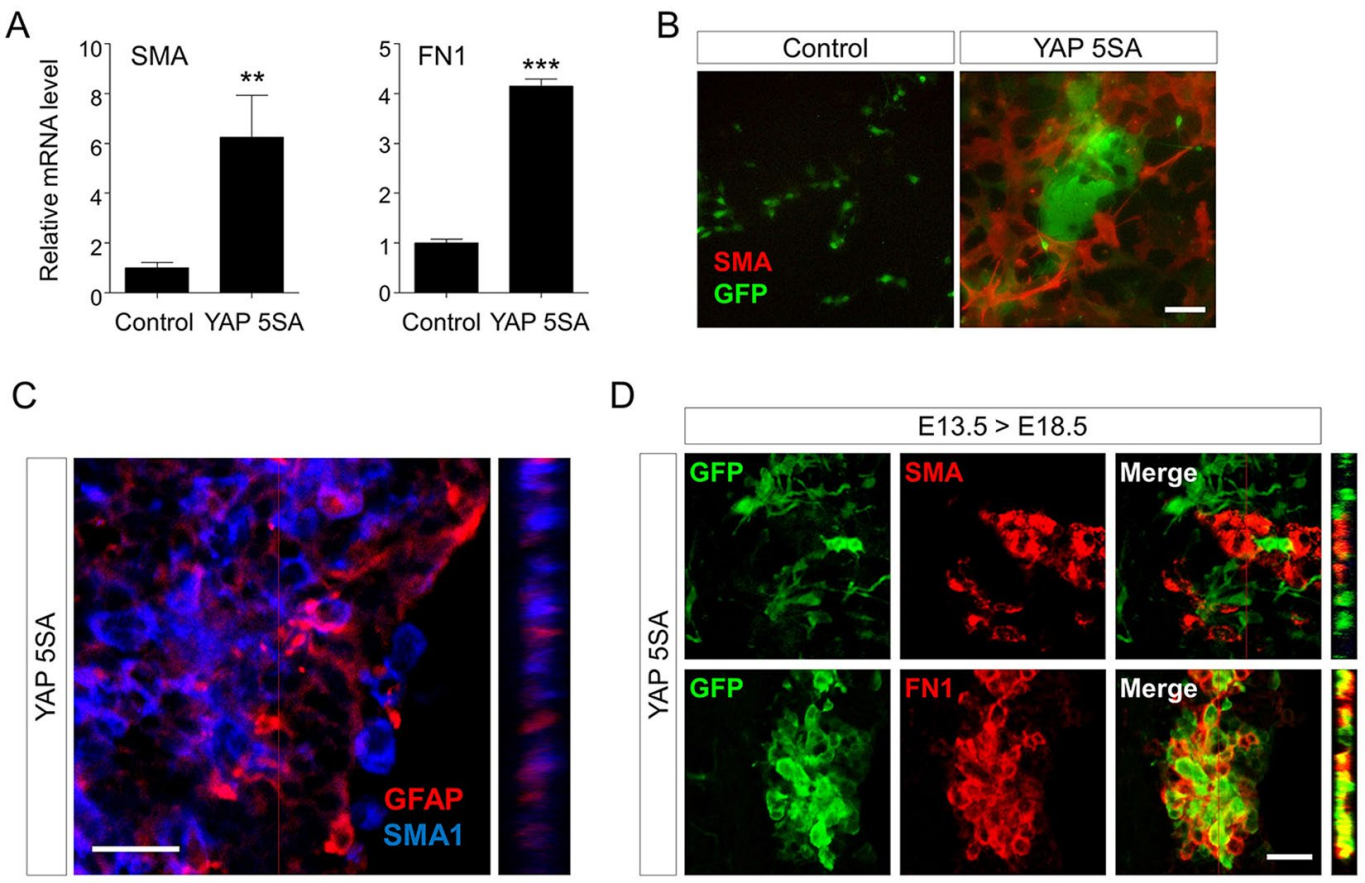
YAP 5SA expression ectopically produces SMA^+^ cells. (**A, B**) E13.5 neural progenitor cells transduced with YAP 5SA retroviruses were cultured in differentiation medium for 2 days, and (**A**) qPCR analysis of SMA and FN1 genes and (**B**) coimmunostaining using anti-GFP (green) and anti-SMA (red) antibodies were carried out. Cortical regions of E18.5 brains transduced with YAP 5SA-expressing retroviruses at E13.5 were labeled with (**C**) anti-GFAP (red) and anti-SMA (blue) antibodies, or (**D**) anti-GFP (green) antibody together with anti-SMA (red) or anti-FN1 (red) antibodies. Z-stack views at the position of the red line are shown on the right. LV, lateral ventricle. Scale bars, 20 μm for (**C**), 50 μm for (**D**), and 100 μm for (**B**). Student’s *t*-test was used to determine statistical significance. **P < 0.01, ***P < 0.001; n ≥ 3.

Next, we attempted to determine the cell type of YAP 5SA-expressing cells. We focused on a report in which cells differentiated from human glioma cells by activation of the YAP paralog, TAZ, were smooth muscle α-actin-positive (SMA^+^) cells^32^. This report defined the SMA^+^ cells as mesenchymal cells and led us to ask whether YAP 5SA-expressing cells are also mesenchymal cells. As shown in Fig. 7A, qPCR analysis showed that YAP 5SA expression increased gene expression of SMA and FN1, both mesenchymal markers, under the *in vitro* neural progenitor differentiation conditions. Immunostaining also revealed massive SMA^+^ cell production *in vitro* (Fig. 7B) and *in vivo* (Fig. 7D). However, in both experimental settings, YAP 5SA-expressing cells were not labeled with anti-SMA antibody, and GFAP^+^ cells also were not SMA^+^ (Fig. 7C). However, instead, YAP 5SA^+^ cells were revealed to be FN1^+^ (Fig. 7D). These results suggest that YAP 5SA expression can induce ectopic FN1^+^ and SMA^+^ mesenchymal cell generation from neural progenitor cells in a cell autonomous- and a non-cell autonomous manner, respectively.

## Discussion

In this study, we show that constitutive YAP activation in neural progenitor cells can induce astrocytic and mesenchymal differentiation in the developing brain in a non-cell autonomous fashion. We also identified CNTF as a novel YAP-regulated gene which is responsible for productive astrogenesis caused by YAP activation.

Recently, Huang *et al*., reported that YAP promotes astrocytic differentiation using conditional YAP knockout mice^24^. However, since they utilized a genetically-engineered mouse model in which all cells with Nestin promoter activity driving conditional Cre recombinase expression starting at E10.5 are equally affected, it was unknown whether YAP-dependent astrogenesis occurs in a cell autonomous or non-cell autonomous manner. In addition, because of the long time interval between the day of YAP deletion (E10.5) and the day of observation (postnatal day 0, P0), this study did not rule out the possibility that YAP depletion and reduced astrogenesis were not in a direct causal relationship. Their results also were not complemented with YAP gain-of-function experiments. We attempted to address these issues by employing an *in utero* retroviral gene delivery method. This approach let us induce YAP overexpression and also track the behaviors of individual YAP-activated cells in a wild-type background.

YAP activation produced GFAP^+^ cells not at E16.5, but at E18.5. In mouse brains, cortical astrogenesis begins after neurogenic periods and peaks around birth^33^. These facts imply that YAP activation itself might not directly instruct astroglial cell fate decision of neural stem cells in the middle of neurogenic periods, but greatly facilitate the onset of differentiation of neural stem cells which are already intrinsically programmed to be astrocytes at late developmental stages. It can be explained in terms of expression of, or responsiveness to, astrogenic ligands. According to our observations, YAP 5SA appears to exert its astrogenesis-inducing effects through the expression of secretory molecules such as CNTF and BMP4. Therefore, it is likely that YAP 5SA could not cause astrogenesis at E16.5 because genes encoding the astrogenic secretory factors were not epigenetically active and accessible until the astrogenic period begins^34^. It is also possible that neural progenitor cells at E16.5 were not ready to respond to astrogenic factors due to a lack of a cellular factor required for astrogenesis. However, *in vitro* differentiation assay showing massive production of astrocytes from E13.5 neural progenitor cells (Fig. 3A) may exclude this possibility.

In terms of astrogenesis, YAP is a unique protein in that it has both cell-autonomous and non-cell autonomous roles. In this study, we focused on CNTF to explain the novel non-cell autonomous effect of YAP because this soluble ligand protein has so far not been implicated in YAP/TAZ-caused cellular events. Among well-known YAP/TAZ target genes, BMP4^31^ and CTGF^29^ have also been reported to increase astrogenesis^35,36^. Thus, it is noteworthy that the YAP/TAZ pathway induces expression of multiple transcriptional target genes encoding astrogenic ligands. Interestingly, as a cell-autonomous factor, YAP also contributes to astrocyte generation. After direct binding to Smad1, a well-known astrogenic transcription factor^37,38^, YAP proteins exert astrogenesis-inducing effects via stabilizing Smad1^24,25^. Thus, the YAP protein itself, and not its target gene products, directly contributes to the cell-autonomous induction of astrogenesis.

Interestingly, in an *in vivo* context, similar observations of productive astrogenesis were not made with wild-type YAP overexpression. We hypothesized that the high levels of YAP-phosphorylating activity of the upstream Hippo signaling kinase cascade in the developing brain might prevent the overexpressed wild-type YAP proteins from entering the nucleus, and cause its degradation. Indeed, immunofluorescence assays revealed that endogenous YAP proteins exist as a phosphorylated form in the VZ, and the amounts of exogenously overexpressed wild-type YAP proteins were considerably low or undetectable even in successfully transduced cells, collectively indicating very unfavorable conditions for YAP activation in the VZ. Cell contact triggers Hippo kinase activation leading to phosphorylation and subsequent degradation of YAP proteins^26^. Thus, the tight cell organization in the VZ of the developing brain is assumed to maintain Hippo kinase activity at a high level. Unlike the *in vivo* experiments, *in vitro* differentiation assay showed a modest increase in astrocyte production by wild-type YAP overexpression (data not shown), but not as strong an effect as seen by YAP 5SA overexpression, probably due to the nature of a less dense *in vitro* cell culture suppressing Hippo kinase activation.

Determination of the cell type generated upon constitutive YAP activation in the VZ at late developmental stages was the most difficult problem in this study. Substantially higher than physiological levels of YAP activation induced by non-degradable YAP proteins is presumed to produce a non-neural cell type which is not present in the developing brain. We first suspected a SMA^+^ mesenchymal cell type based on a report showing that a constitutively active form of TAZ generates SMA^+^ mesenchymal cells from malignant glioma in a TEAD-dependent fashion^32^. At first glance, our observations appeared to be in accord with their findings, but there was a profound difference in the way of mesenchymal cell induction. The TAZ/TEAD complex was shown to increase SMA and mesenchymal gene transcription by directly activating their promoters, meaning cell autonomous effects of TAZ, whereas our *in vitro* and *in vivo* immunostaining results of non-overlapping expression patterns of SMA and YAP 5SA suggest a non-cell autonomous role for YAP in the SMA^+^ cell production. Although the results are not directly comparable due to different experimental settings, these observations may constitute additional valuable evidence that YAP and TAZ actions are not completely identical in all contexts. Finally, we were able to identify the YAP 5SA-expressing cells as FN1^+^, and these findings raise two interesting hypotheses: First, FN1^+^ mesenchymal cells are distinct from SMA^+^ mesenchymal cells. Second, FN1^+^ mesenchymal cells can induce SMA^+^ mesenchymal cells.

A number of studies have revealed a close relationship between normal stem cells and cancer stem cells in various aspects. Particularly in the brain, brain cancer stem cells were found to share many similarities with normal neural stem cells^39,40^. Considering that YAP proteins are readily detected in adult brain tumors^41^, our observations that neural stem cells with high levels of YAP activity can produce GFAP^+^ cells have important implications in brain tumor progression. GFAP^+^ astrocytes are frequently found in the vicinity of glioma cells, and are reported to be associated with tumor malignancy^42^. For example, astrocytes were found to be closely linked with increasing grade of PDGF-induced gliomas^43^. Furthermore, several reports have provided a molecular basis for the role of astrocytes in the aggressive behavior of brain cancer cells. Astrocytes enhance glioblastoma cell invasion via activation of metalloproteinases^42,44^ and neurotrophic factors^45^. Astrocytes also can induce glioblastoma cell proliferation by secreting stromal cell-derived factor-1 (SDF-1)^46,47^. These studies indicate that astrocytes play an important role in promoting tumor progression, and our report provides a molecular mechanism by which GFAP^+^ cells can be generated from brain tumors. In addition, we would like to emphasis that our study, for the first time to our knowledge, showed that the expression of CNTF which was previously identified as an important inducer of reactive gliosis^48,49^, was greatly increased in the YAP-activated cells. However, it is not clear whether CNTF is a direct transcriptional target of YAP. According to our detailed *in silico* CNTF promoter examination and a luciferase assay reporting CNTF promoter activity, CNTF is not likely to be a direct downstream target of YAP (data not shown).

As mentioned, the unexpected interesting findings in this study are that YAP activation can induce ectopic FN1^+^ (cell autonomously) and SMA^+^ (non-cell autonomously) mesenchymal cell differentiation in the embryonic brain context. It would be also plausible to assume that YAP activation can drive mesenchymal cell differentiation, like astrocytic differentiation, from brain cancer stem cells in the adult. Given that mesenchymal transition in glioblastoma was reported to be associated with poor response to radiation and shorter survival in patients with glioblastoma^50^, our study has another important implication for understanding the role of YAP in brain tumor progression. Although transcription factors, C/EBPβ, STAT3, and TAZ, have been shown to be able to induce mesenchymal transition in glioblastoma^32,51^, it has been unknown whether these factors trigger the mesenchymal transition in a cell autonomous manner or by indirectly modifying the tumor microenvironment. Our analyses add interesting evidence supporting the latter by showing that YAP activation can promote mesenchymal cell production but that the YAP-activated cells themselves are not mesenchymal cells. Collectively, in addition to the well-known ability of YAP to increase cell proliferation by enhancing cell cycle-promoting gene expression^52^, YAP may contribute to cancer malignancy in two indirect and independent ways based on our findings: induction of GFAP^+^ astrocytes and mesenchymal cell generation. Further studies are needed to clarify this hypothesis using brain tumor models.

## Materials and Methods

### Plasmids

The wild-type and constitutively active mutant (YAP 5SA) YAP vectors were purchased from Addgene (plasmids #33091 and #33093, respectively) (Cambridge, MA), and subcloned into the MluI site of the retroviral vector MSIG^27^. The YAP 5SA∆PDZ, S94A, WW1* and WW2* mutants were generated by site-directed mutagenesis using the following primers: ∆PDZ (5′-CCAAGCTAGATAAAGAAAGCTAGTTAATACGCGTGGGCCCGCGG-3′), S94A (5′-GGCTCCGGAAGCTGCCCGACGCCTTCTTCAAGCCGCCGGAG-3′), WW1* (5′- ACATCGATCAGACAACAACAGCCCAGGACGCCAGGAAGGCCATGCTGTCCCA-3′), WW2* (5′-ATAAGAACAAGACCACCTCTGCCCAGACGCCAGGCTTGACCCTCGTTTTGC-3′) and the complementary reverse primers. The YAP 5SA WW1/2* mutant was generated using the WW2* primer set with the YAP 5SA WW1* template.

### Retroviral vector preparation

The method of retroviral vector production has been previously described^53^. Briefly, the retroviral construct was transfected into HEK 293 T cells with a gag-pol (pCA-gag-pol) and env-expressing vector (VSV-G) using polyethyleneimine (Sigma, St. louis, MO). The supernatant was collected 48 h after transfection, filtered through a 0.45 μm filter and frozen at −80 °C until used. Concentrated viral stocks were prepared by ultracentrifugation at 25,000 rpm for 90 min at 4 °C in an SW28 rotor (Beckman Coulter, Palo Alto, CA). Pellets were resuspended in 50 μl of PBS at 4 °C for about 12 h, and virus aliquots stored at −80 °C.

### Animals and *in utero* injection

All animal procedures were approved by the Institutional Animal Care and Use Committee of Sungkyunkwan University under the animal protocol No. 2016-04-0005-2. All experiments were conducted in accordance with the institutional guidelines and regulations for animal experiments. Timed pregnant CD1 mice (Orient Bio, Osan, Korea) were used for viral injections, and embryos were considered 0.5-days old (embryonic day 0.5, E0.5) when a vaginal plug was detected in the morning. Prior to injection, pregnant mice were anesthetized with pentobarbital sodium (Hanlim Pharm, Gyeonggi, Korea) and ultracentrifuge-concentrated viruses containing polybrene (final concentration 80 μg/ml) were injected into the telencephalic ventricle of E13.5 embryos.

### Immunofluorescence

Standard immunofluorescence procedures were used for visualization of target gene expression in retroviral vector-injected animals. Briefly, gene-transferred brains at E16.5 or E18.5 were fixed in 4% paraformaldehyde and cryosectioned. Sections were washed in PBS, then blocked for 1 h with PBS containing 10% fetal bovine serum and 0.2% Triton X-100. Samples were then incubated overnight at 4 °C with anti-GFP (Invitrogen, Carlsbad, CA, A-11122), anti-SOX2 (Millipore, Temecula, CA, ab5603), anti-Dcx (Abcam, Cambridge, MA, ab18723), anti-GFAP (Cell Signaling Technology, Beverly, MA, 3670 S), anti-GFAP (Millipore, AB5541, used only for Fig. 7C), anti-CNTF (Peprotech, Rocky Hill, NJ, 500-P140), anti-Fibronectin1 (ABclonal, Wuhan, China, WH130143) and anti-SMA (Santa Cruz Biotechnology, Santa Cruz, CA, sc53142) primary antibodies, washed three times in PBS and incubated for 1 h at room temperature with Alexa Fluor-conjugated secondary antibodies (Invitrogen, A-31570, A-21206, A-31572) diluted in blocking solution. Samples were further counterstained with 4′,6-diamidino-2-phenylindole (DAPI) (Sigma, D9541). Images were acquired using a Zeiss LSM 700 confocal microscope (Oberkochen, Germany). The z-stack images used in Figs. 2D, 5C, 6D and 7D comprised 15 axial positions spanning a total range of 14 μm.

### *In vitro* differentiation of neural progenitor cells

Primary neural progenitor cells were prepared from E13.5 embryo brains. Dissected brain tissue was minced, washed thrice with PBS, and incubated in 0.25% trypsin (Invitrogen) at 37 °C for 5 min. DNase and ovomucoid trypsin inhibitor (Worthington, Freehold, NJ) were added, and samples were triturated using a fire-polished Pasteur pipette. Cells were washed twice with DMEM/F12 media, resuspended in PBS, and ran through a 40 µm cell strainer (Falcon, Franklin Lakes, NJ). For the *in vitro* co-differentiation assay, E13.5 primary neural progenitors were transduced with concentrated retroviral vectors expressing YAP5SA and mixed with untransduced E13.5 neural progenitor cells at a ratio of 1:5. The mixed cells were then seeded on poly-L-ornithine (PLO)- and laminin (LM)-coated plates and cultured in differentiation media (DMEM/F12 containing 1% FBS) for 3 days. To prepare conditioned media, retrovirally transduced E13.5 neural progenitor cells were plated on PLO/LM-coated plates and incubated in fresh differentiation media. Conditioned media was harvested at day 3 from the culture. To induce differentiation with conditioned medium, E13.5 untransduced neural progenitor cells were seeded on PLO/LM-coated plates and incubated in differentiation media composed of fresh- and conditioned media prepared from YAP 5SA culture at a volume ratio of 1:1. Heat inactivation was done by incubation of conditioned media at 56 °C for 30 min. To neutralize CNTF, differentiation medium were pretreated with 10 µg/ml anti-CNTF antibody (Peprotech, 500-P140) at 37 °C for 2 h. For double immunostaining, differentiated cells were fixed with 4% paraformaldehyde for 15 min and ice-cold ethanol for 90 sec and washed twice with PBS. For primary antibodies, we used antibodies to GFP (Invitrogen, A-11122), GFAP (Cell Signaling Technology, 3670 S) and SMA (Santa Cruz Biotechnology, sc53142). Fluorescent images were obtained with an upright microscope (Eclipse 80i; Nikon, Tokyo, Japan).

### Statistical analysis

Statistical tests were performed using SPSS (statistical product and service solution software, IBM) version 24. Statistical differences were calculated by using unpaired two-tailed Student’s t-test. Significance was established at *P* < 0.05. All data represent three or more independent experiments.

## Supplementary information

Supplementary information

## References

[1] Piet R, Vargová L, Syková E, Poulain DA, Oliet SH (2004). Physiological contribution of the astrocytic environment of neurons to intersynaptic crosstalk. Proc. Natl. Acad. Sci. USA.

[2] Parri R, Crunelli V (2003). An astrocyte bridge from synapse to blood flow. Nat. Neurosci..

[3] Alvarez JI, Katayama T, Prat A (2013). Glial influence on the blood brain barrier. Glia.

[4] Han X (2013). Forebrain engraftment by human glial progenitor cells enhances synaptic plasticity and learning in adult mice. Cell Stem Cell.

[5] Anderson MA (2016). Astrocyte scar formation aids central nervous system axon regeneration. Nature.

[6] Barker AJ, Ullian EM (2008). New roles for astrocytes in developing synaptic circuits. Commun. Integr. Biol..

[7] Sloan SA, Barres BA (2014). Mechanisms of astrocyte development and their contributions to neurodevelopmental disorders. Curr Opin. Neurobiol..

[8] Ge W-P, Miyawaki A, Gage FH, Jan YN, Jan LY (2012). Local generation of glia is a major astrocyte source in postnatal cortex. Nature.

[9] Ochiai W, Yanagisawa M, Takizawa T, Nakashima K, Taga T (2001). Astrocyte differentiation of fetal neuroepithelial cells involving cardiotrophin-1-induced activation of STAT3. Cytokine.

[10] Nakashima K, Yanagisawa M, Arakawa H, Taga T (1999). Astrocyte differentiation mediated by LIF in cooperation with BMP2. FEBS. Lett..

[11] Rajan P, McKay RD (1998). Multiple routes to astrocytic differentiation in the CNS. J. Neurosci..

[12] Derouet D (2004). Neuropoietin, a new IL-6-related cytokine signaling through the ciliary neurotrophic factor receptor. Proc. Natl. Acad. Sci. USA..

[13] Uemura A (2002). Cardiotrophin-like cytokine induces astrocyte differentiation of fetal neuroepithelial cells via activation of STAT3. Cytokine.

[14] Miyazono K, Kusanagi K, Inoue H (2001). Divergence and convergence of TGF‐β/BMP signaling. J Cell Physiol.

[15] Lee J, Borboa AK, Baird A, Eliceiri BP (2011). Non-invasive quantification of brain tumor-induced astrogliosis. BMC. Neurosci..

[16] O’Callaghan JP, Kelly KA, VanGilder RL, Sofroniew MV, Miller DB (2014). Early activation of STAT3 regulates reactive astrogliosis induced by diverse forms of neurotoxicity. PLoS One.

[17] Yu Z, Yu P, Chen H, Geller HM (2014). Targeted inhibition of KCa3. 1 attenuates TGF‐β‐induced reactive astrogliosis through the Smad2/3 signaling pathway. J. Neurochem..

[18] Saucedo LJ, Edgar BA (2007). Filling out the Hippo pathway. Nat. Rev. Mol. Cell. Biol..

[19] Hilman D, Gat U (2011). The evolutionary history of YAP and the hippo/YAP pathway. Mol Biol Evol.

[20] Kanai F (2000). TAZ: a novel transcriptional co-activator regulated by interactions with 14‐3‐3 and PDZ domain proteins. EMBO J.

[21] Cao X, Pfaff SL, Gage FH (2008). YAP regulates neural progenitor cell number via the TEA domain transcription factor. Genes. Dev..

[22] Gee ST, Milgram SL, Kramer KL, Conlon FL, Moody SA (2011). Yes-associated protein 65 (YAP) expands neural progenitors and regulates Pax3 expression in the neural plate border zone. PloS One.

[23] Lavado A (2013). Tumor suppressor Nf2 limits expansion of the neural progenitor pool by inhibiting Yap/Taz transcriptional coactivators. Development.

[24] Huang Z (2016). YAP stabilizes SMAD1 and promotes BMP2-induced neocortical astrocytic differentiation. Development.

[25] Huang Z (2016). Neogenin promotes BMP2 activation of YAP and Smad1 and enhances astrocytic differentiation in developing mouse neocortex. J. Neurosci..

[26] Zhao B (2007). Inactivation of YAP oncoprotein by the Hippo pathway is involved in cell contact inhibition and tissue growth control. Genes. Dev..

[27] Byun S-H (2017). TRBP maintains mammalian embryonic neural stem cell properties by acting as a novel transcriptional coactivator of the Notch signaling pathway. Development.

[28] Oka T (2010). Functional complexes between YAP2 and ZO-2 are PDZ domain-dependent, and regulate YAP2 nuclear localization and signalling. Biochem. J.

[29] Zhao B (2008). TEAD mediates YAP-dependent gene induction and growth control. Genes. Dev..

[30] Oka T, Mazack V, Sudol M (2008). Mst2 and Lats kinases regulate apoptotic function of Yes kinase-associated protein (YAP). J Biol Chem.

[31] Lai D, Yang X (2013). BMP4 is a novel transcriptional target and mediator of mammary cell migration downstream of the Hippo pathway component TAZ. Cell Signal.

[32] Bhat KP (2011). The transcriptional coactivator TAZ regulates mesenchymal differentiation in malignant glioma. Genes. Dev..

[33] Skoff RP, Knapp PE (1991). Division of astroblasts and oligodendroblasts in postnatal rodent brain: evidence for separate astrocyte and oligodendrocyte lineages. Glia.

[34] Mallamaci A (2013). Developmental control of cortico-cerebral astrogenesis. Int. J. Dev. Biol..

[35] Mendes F (2015). Connective-tissue growth factor (CTGF/CCN2) induces astrogenesis and fibronectin expression of embryonic neural cells *in vitro*. PLoS One.

[36] Cole, A. E., Murray, S. S. & Xiao, J. Bone morphogenetic protein 4 signalling in neural stem and progenitor cells during development and after Injury. Stem Cells Int 2016, 10.1155/2016/9260592 (2016). 10.1155/2016/9260592 PMC4884839 27293450

[37] Nakashima K (1999). Synergistic signaling in fetal brain by STAT3-Smad1 complex bridged by p300. Science.

[38] Fukuda S (2007). Potentiation of astrogliogenesis by STAT3-mediated activation of bone morphogenetic protein-Smad signaling in neural stem cells. Mol. Cell. Biol..

[39] Ignatova TN (2002). Human cortical glial tumors contain neural stem‐like cells expressing astroglial and neuronal markers *in vitro*. Glia.

[40] Hemmati HD (2003). Cancerous stem cells can arise from pediatric brain tumors. Proc. Natl. Acad. Sci. USA..

[41] Orr BA (2011). Yes-associated protein 1 is widely expressed in human brain tumors and promotes glioblastoma growth. J. Neuropathol. Exp. Neurol..

[42] Le DM (2003). Exploitation of astrocytes by glioma cells to facilitate invasiveness: a mechanism involving matrix metalloproteinase-2 and the urokinase-type plasminogen activator–plasmin cascade. J. Neurosci..

[43] Becher OJ (2008). Gli activity correlates with tumor grade in platelet-derived growth factor–induced gliomas. Cancer Res..

[44] Liu L (2010). Astrocyte elevated gene-1 upregulates matrix metalloproteinase-9 and induces human glioma invasion. Cancer Res..

[45] Hoelzinger DB, Demuth T, Berens ME (2007). Autocrine factors that sustain glioma invasion and paracrine biology in the brain microenvironment. J. Natl. Cancer Inst..

[46] Bajetto A (1999). Glial and neuronal cells express functional chemokine receptor CXCR4 and its natural ligand stromal cell-derived factor 1. J. Neurochem.

[47] Barbero S (2002). Expression of the chemokine receptor CXCR4 and its ligand stromal cell‐derived factor 1 in human brain tumors and their involvement in glial proliferation *in vitro*. Ann. N. Y. Acad. Sci..

[48] Kahn M, Ellison J, Speight G, De Vellis J (1995). CNTF regulation of astrogliosis and the activation of microglia in the developing rat central nervous system. Brain Res..

[49] Winter CG, Saotome Y, Levison SW, Hirsh D (1995). A role for ciliary neurotrophic factor as an inducer of reactive gliosis, the glial response to central nervous system injury. Proc. Natl. Acad. Sci. USA..

[50] Bhat KP (2013). Mesenchymal differentiation mediated by NF-κB promotes radiation resistance in glioblastoma. Cancer Cell..

[51] Carro MS (2010). The transcriptional network for mesenchymal transformation of brain tumours. Nature.

[52] Mizuno T (2012). YAP induces malignant mesothelioma cell proliferation by upregulating transcription of cell cycle-promoting genes. Oncogene.

[53] Han D (2017). Human cytomegalovirus IE2 protein disturbs brain development by the dysregulation of neural stem cell maintenance and the polarization of migrating neurons. J. Virol.

