# Supplementary material for: Non-cell autonomous promotion of astrogenesis at late embryonic stages by constitutive YAP activation
Source: Sci Rep. 2020 Apr 27;10:7041. doi: 10.1038/s41598-020-63890-z (PMC7184574; doi:10.1038/s41598-020-63890-z)

Supplementary Information

Non-cell autonomous promotion of astrogenesis at late embryonic stages by constitutive YAP activation

Dasol Han, Mookwang Kwon, Sun Min Lee, Samuel J. Pleasure, and

Keejung Yoon

Fig. 4D

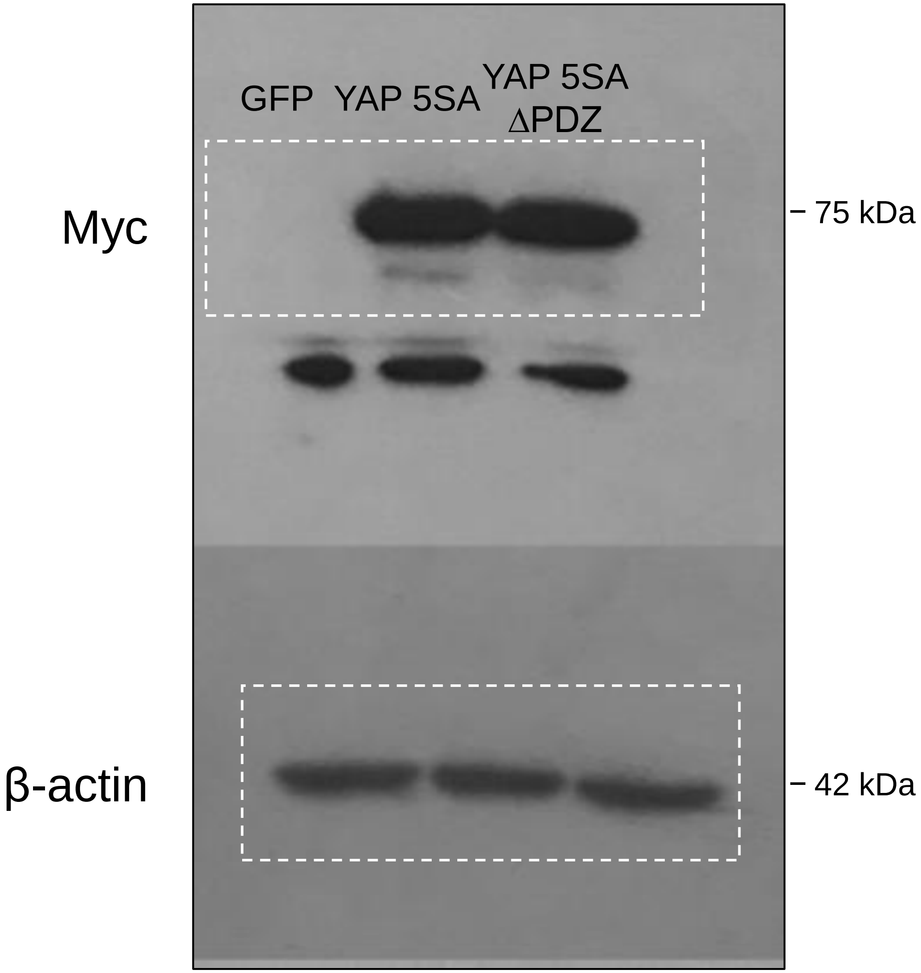

Fig. 5B

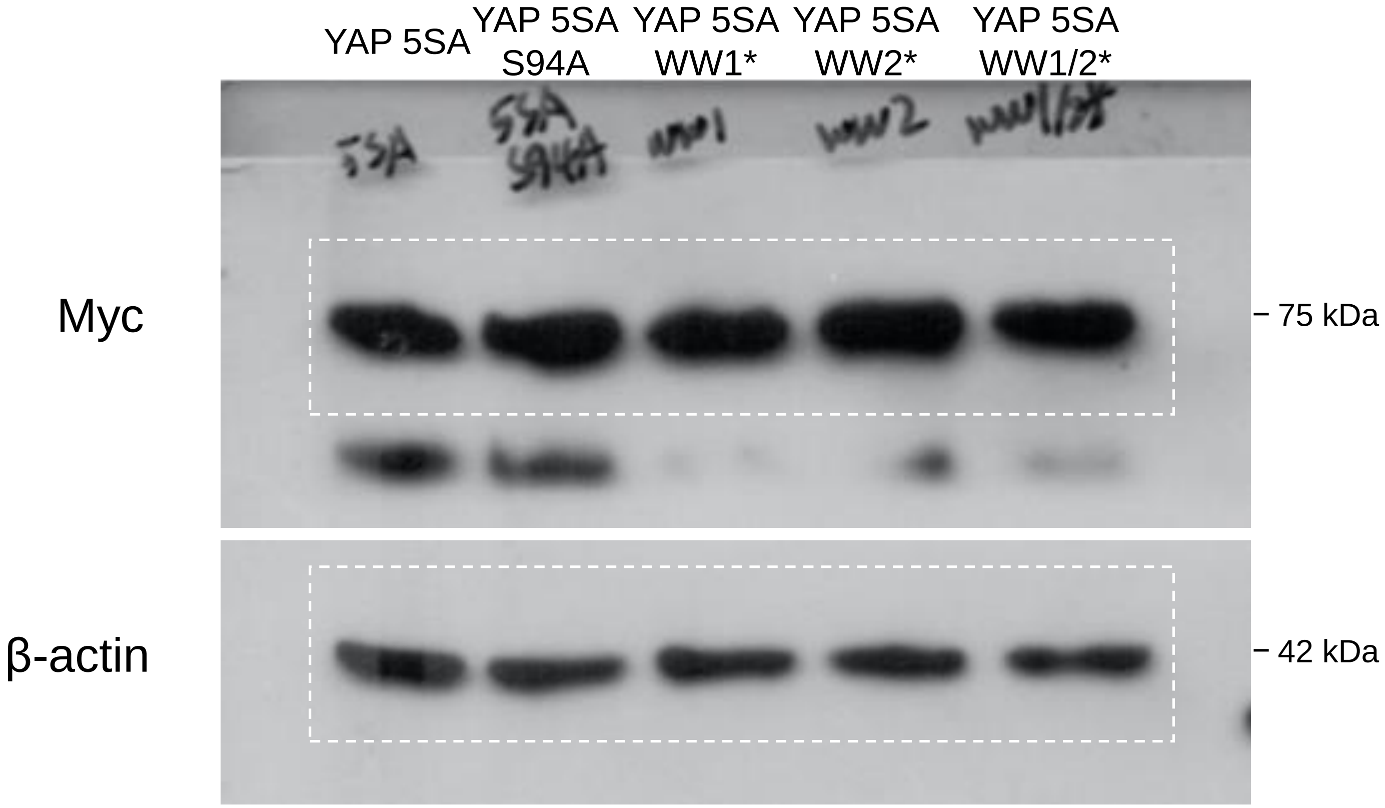
YAP 5SA
YAP 5SA S94A
YAP 5SA WW1*
YAP 5SA WW2*
YAP 5SA WW1/2*
Myc
β-actin
75 kDa
42 kDa

Fig. 6B

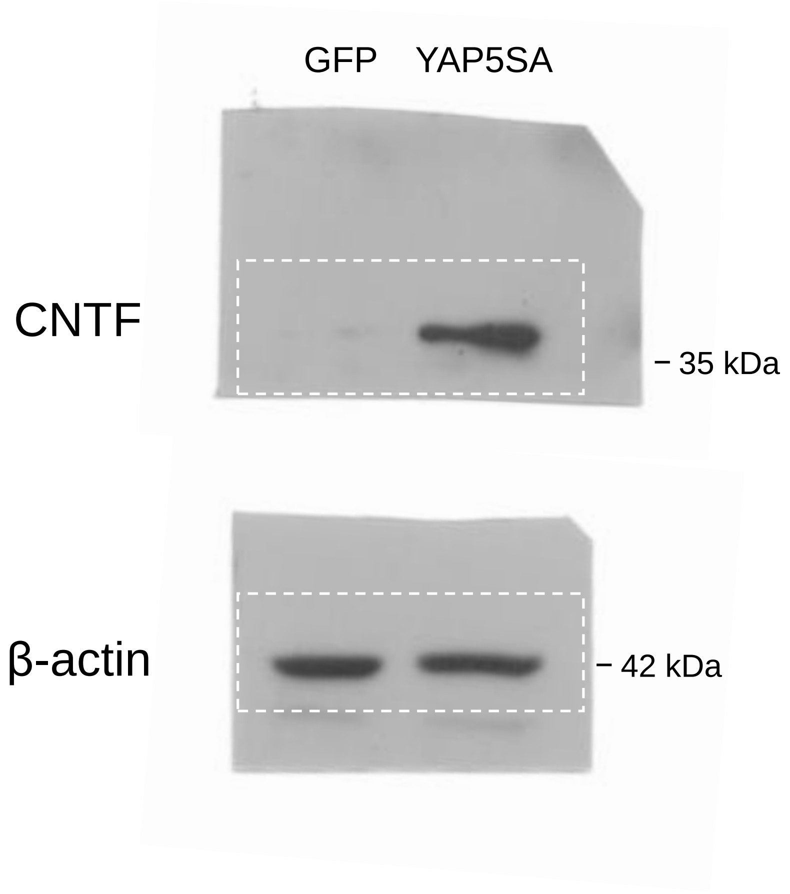

Supplement: Supplementary file 1 — Supplementary information [file 41598_2020_63890_MOESM1_ESM.pdf]
